# Optimizing the Synthesis of Core/shell Structure Au@Cu_2_S Nanocrystals as Contrast-enhanced for Bioimaging Detection

**DOI:** 10.1038/s41598-018-27015-x

**Published:** 2018-06-11

**Authors:** Liwei Liu, Siyi Hu, Yue Wang, Shaozhuang Yang, Junle Qu

**Affiliations:** 1 Key Laboratory of Optoelectronic Devices and Systems of Guangdong Province, College of Optoelectronic Engineering, Shenzhen University, Shenzhen, Guangdong Province 518060 P.R. China; 2 CAS Key Laboratory of Bio-Medical Diagnostics, Suzhou Institute of Biomedical Engineering and Technology, Chinese Academy of Sciences, Suzhou, Jiangsu 215163 P.R. China; 3 International Joint Research Center for Nanophotonics and Biophotonics, School of Science, Changchun University of Science and Technology, Changchun, Jilin Province 130022 P.R. China

## Abstract

In this paper, we reported Au@Cu_2_S nanocrystals in the aqueous phase with a core/shell structure and dBSA encapsulation. The dBSA-Au@Cu_2_S crystals formed with an average size of approximately 9 nm. There was a strong absorption in the near-infrared (NIR) field located at 1348 nm, and they exhibited low toxicity in the *in vitro* tests. Furthermore, we demonstrated that dBSA-Au@Cu_2_S could be used for optical coherence tomography (OCT). The *in vivo* experimental results show that the OCT signal increased as the concentration of nanocrystals increased. In this research, we revealed that these core/shell-structured nanocrystals along with their low toxicity and excellent biocompatibility could be a valuable tool for current and future contrast-enhanced *in vivo* studies.

## Introduction

The development of synthetic nanocrystals has facilitated research on the strong absorption behaviours of metal nanocrystals. Some researchers have studied the effects and mechanisms of the strong NIR absorption behaviours of Cu_2_S and complex Cu_x_S_x_ and Cu_X_S nanocrystals^1–4^. There has been an emphasis on understanding the physics of charge carrier interactions and the optical characteristics, and developing biological therapeutic agents, biosensors, and optoelectronic devices. Recently, copper sulphide nanocrystals as metal semiconductor nanocrystals have attracted more attention due to their low cost, non-toxicity and strong absorption in the near-infrared (NIR). It is especially prevalent for complex structures combining two or more different metal materials to show enhanced physical, optical and chemical properties. Thus, this enabled them to serve as a good candidate in optoelectronics, photochemistry and biological therapeutic agent applications^5–8^.

Cu_2_S, an important p-type semiconductor nanocrystal with a bulk band gap of 1.21 eV, has been investigated as a metal material since the first report of Cu_2_S nanocrystals in 1998^9–12^. Significant advantages, such as strong absorption, photostability and low toxicity, have been demonstrated^13,14^. Recently, Cu_2_S along with other complex-structured semiconductor composite nanocrystals have been reported as well. There is no doubt that Cu_2_S nanocrystals will offer a new tool for multiplex nanocrystals synthesis and applications^15–18^.

Au is one of the main materials resulting in nanoplasmonics in semiconductor nanocrystals^19,20^. The Au particles enhance the optical and chemical performance through trapping photogeneration and charge recombination suppression. According to these factors, encapsulating Au with other nanoparticles that are suitable for multiplex nanocrystal studies, such as Cu_2_S, Ag, and others can form complex nanocrystal structures^21–25^.

Furthermore, the formation mechanism of these Au@Cu_2_S nanocrystals have been reported^26–28^. While certain reports have only discussed research pertaining to the optical characteristics and the applications in photocatalysis, solar cells and photochemistry, there has not yet been a report on biological applications, especially in the clinical settings. Nowadays Optical Coherence Tomography (OCT), X-ray Computed Tomography (CT) and Positron Emission Tomography (PET) are widely used in clinical imaging detection setting. In order to improve the resolution, penetration depth and the capability of 3-dimension image reconstruction of these imaging detection setting, some nanocrystals have been used as a contrasts agent in these imaging setting based on their low toxicity, strong absorption and scattering characteristic, especially in OCT imaging study^29–31^. However, as we have known, the OCT image was obtained by the intrinsic scattering and absorption properties of the biological tissues meaning that the OCT imaging signal can be enhanced with extrinsic contrast agents that lead to higher absorption or scattering localized at targeted biological tissues. Through the extrinsic contrast agents with good properties, the quality of OCT imaging can be improved greatly. Therefore, using nanocrystals for contrast enhancement in OCT imaging detection has become a trend^32–34^.

Herein, we report a new method for the synthesis of Au@Cu_2_S nanocrystals in the aqueous phase, using glutathione (GSH) as the surfactant and sulphur source. It was then encapsulated in denatured bovine serum albumin (dBSA) to enhance its biocompatibility *in vitro* and *in vivo*. The cell stability can be maintained at 70% even at a concentration as high as 500 μgml^−1^ for 48 hours. For further *in vivo* detection, we also performed an OCT test. The OCT imaging signals were obtained from dBSA-Au@Cu_2_S nanocrystals at concentrations range from 1 mg/ml to 4 mg/ml in a living mouse. Due to the biocompatibility and low toxicity, these core/shell structured nanocrystals have great potential to serve as a new candidate of therapeutic agents in cancer detection and future contrast-enhanced *in vivo* OCT imaging studies.

## Results and Discussion

**Figure 1:**
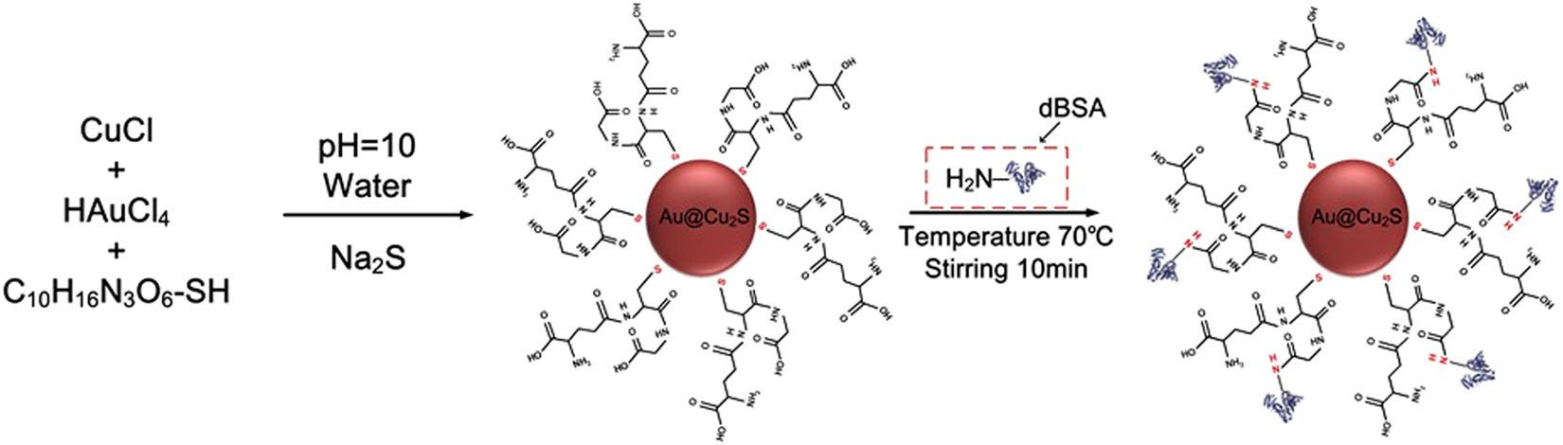
Schematic of synthesis of structures of dBSA-Au@Cu_2_S.

Figure 1 shows a schematic illustration of the synthesis process of Au@Cu_2_S and the conjugating process of dBSA with Au@Cu_2_S.

**Figure 2:**
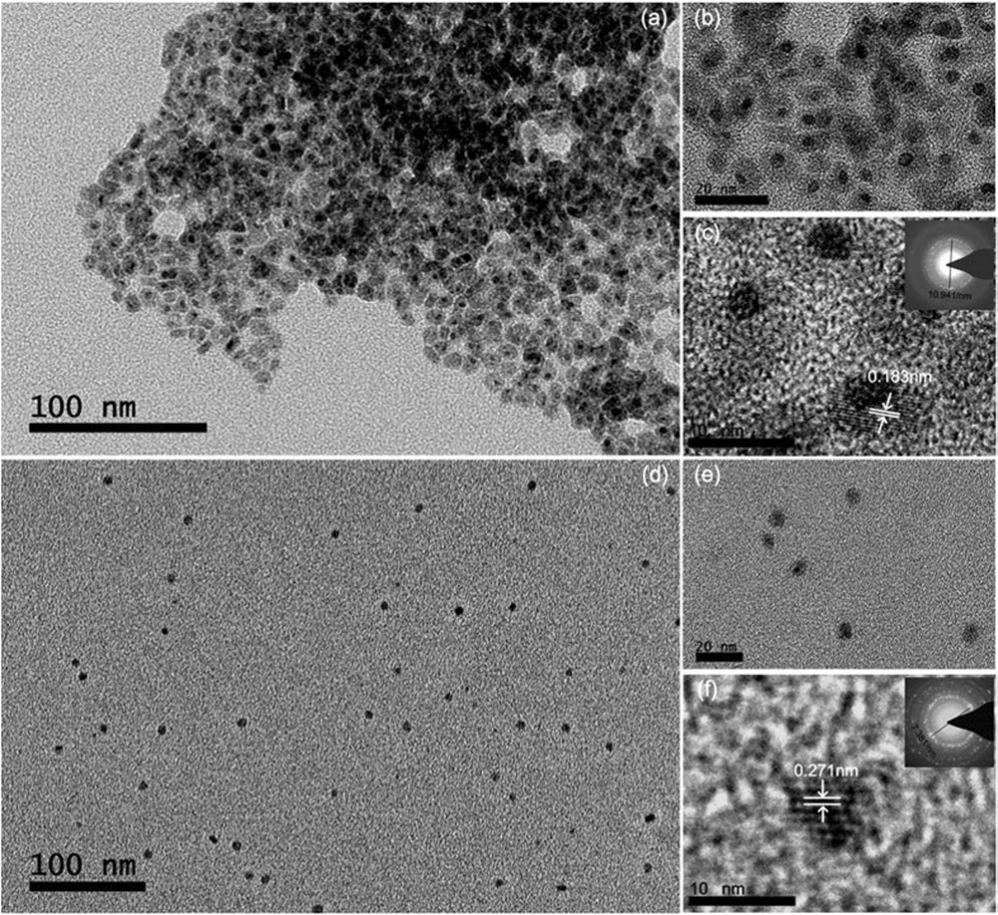
TEM images of (**a**) Au@Cu_2_S and (**d**) dBSA-Au@Cu_2_S. (**b**) and (**c**) are the high resolution TEM images of Au@Cu_2_S, and the insets are the electron diffraction patterns. (**e**) and (**f**) are the high-resolution TEM images of dBSA-Au@Cu_2_S, and the insets are electron diffraction patterns; scale bars are 10, 20 and 100 nm, respectively.

Figure 2 shows the transmission electron microscopy (TEM) images of Au@Cu_2_S and dBSA-Au@Cu_2_S nanocrystals. The image shows that the final products consist of Au@Cu_2_S and dBSA-capped Au@Cu_2_S nanocrystals, respectively. As evidently observed from Fig. 2(a–f), the nanocrystals are sufficiently dispersed and well separated. The size of the two samples is 7 ± 1 nm and 9 ± 3 nm. The high-resolution image shows that the particles are highly crystalline, and the diffraction rings of (c) and (f) inside can be identified at d = 0.195 ± 0.005 and 0.271 ± 0.005 nm, respectively.

We simulated the absorption intensity of Au@Cu_2_S using COMSOL Multiphysics 5.0 software because the simulation of the localized surface plasmon resonance (LSPR) with copper nanoparticles could substitute for the absorption intensity^35^. Therefore, we simulated the absorption intensity by using two equations in the software:

1 $${\sigma }_{{\rm{abs}}}=\frac{1}{{I}_{0}}\iiint Q{\rm{d}}V$$

2 $$\nabla \times {\mu }_{r}^{-1}(\nabla \times \overrightarrow{E})-{k}_{0}^{2}({\varepsilon }_{r}-\frac{j\sigma }{\omega {\varepsilon }_{0}})\overrightarrow{E}=0$$

In Equation 1, $Q$ is the absorption intensity in a single section, which could be shown as ewfd2. Q_h_ in COMSOL, ewfd2 is the electromagnetic waves frequency domain, Q_h_ is the total power dissipation density, and ${\sigma }_{abs}$ is the absorptivity of the materials. In Equation 2, ${\mu }_{r}$ is the relative permeability, ${\varepsilon }_{0}$ is the permittivity of vacuum, ${\varepsilon }_{r}$ is the relative dielectric constant, $\sigma$ is the specific conductance. In Equation 2, ${\varepsilon }_{r}$ could be the expressed as another equation as follows:

3 $${\varepsilon }_{r}={(n-ik)}^{2},\sigma =0,{\mu }_{r}=1$$

Therefore, we need to measure the index of refraction to express the relative dielectric constant. The index of refraction of Au@Cu_2_S nanocrystals was acquired by using Mach-Zehnder Interferometer (MZI)^36^. Therefore, we found the refractive index changed with wavelength, and by using Origin 8.0 software to curve-fitting, the equation could be the expression as

4 $$n={A}_{1}+({A}_{2}-{A}_{1})[\frac{P}{1+{10}^{\mathrm{log}({x}_{1}-\lambda ){h}_{1}}}+\frac{1-P}{1+{10}^{\mathrm{log}({x}_{2}-\lambda ){h}_{2}}}]$$

**Figure 3:**
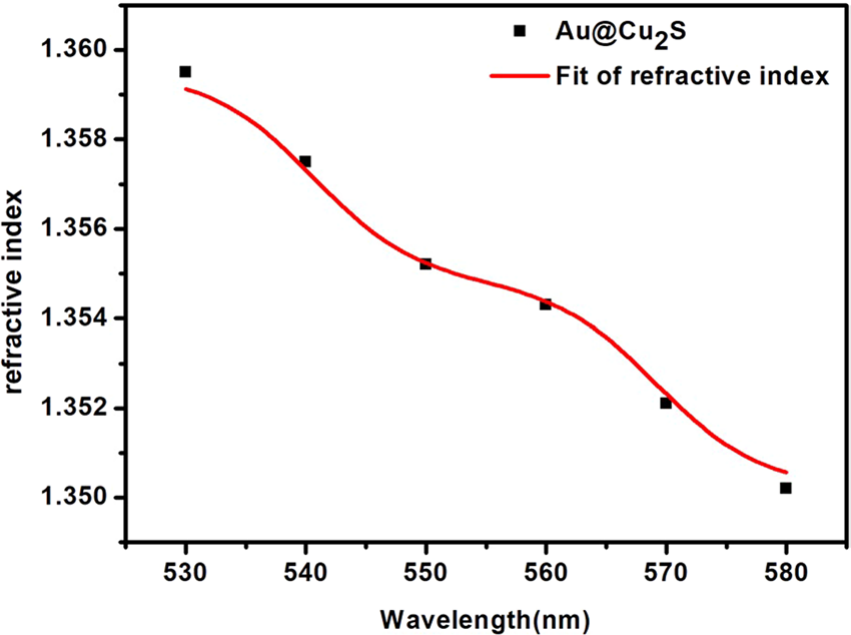
The relationship between refractive index and wavelength of Au@Cu_2_S nanocrystals.

Where *A*_1_ = 1.3502, *A*_2_ = 1.3595, *h*_1_ = −0.1, *h*_2_ = −0.1, *P* = 0.505, ${\mathrm{log}}^{{x}_{1}}$ = 540.65217, ${\mathrm{log}}^{{x}_{2}}$ = 569.31818, $n$ is the refractive index of Au@Cu_2_S nanocrystals, $\lambda$ is the wavelength. We applied the Maher Zed interferometer to test materials refractive with the different wavelength. Then we input the data of materials refraction in different wavelength into the Origin 8.0 software, the numerical curve fitting results using the function BiDoseResp option. All of the parameters, Y for materials refraction, X for wavelength, the rest of the parameters (*A*_1_, *A*_2_, ect.) are constants by BiDoseResp function, for the software to BiDoseResp function are fixed values with no special physical meaning. The relationship between refractive index and wavelength of Au@Cu_2_S nanocrystals, as shown in Fig. 3.

**Figure 4:**
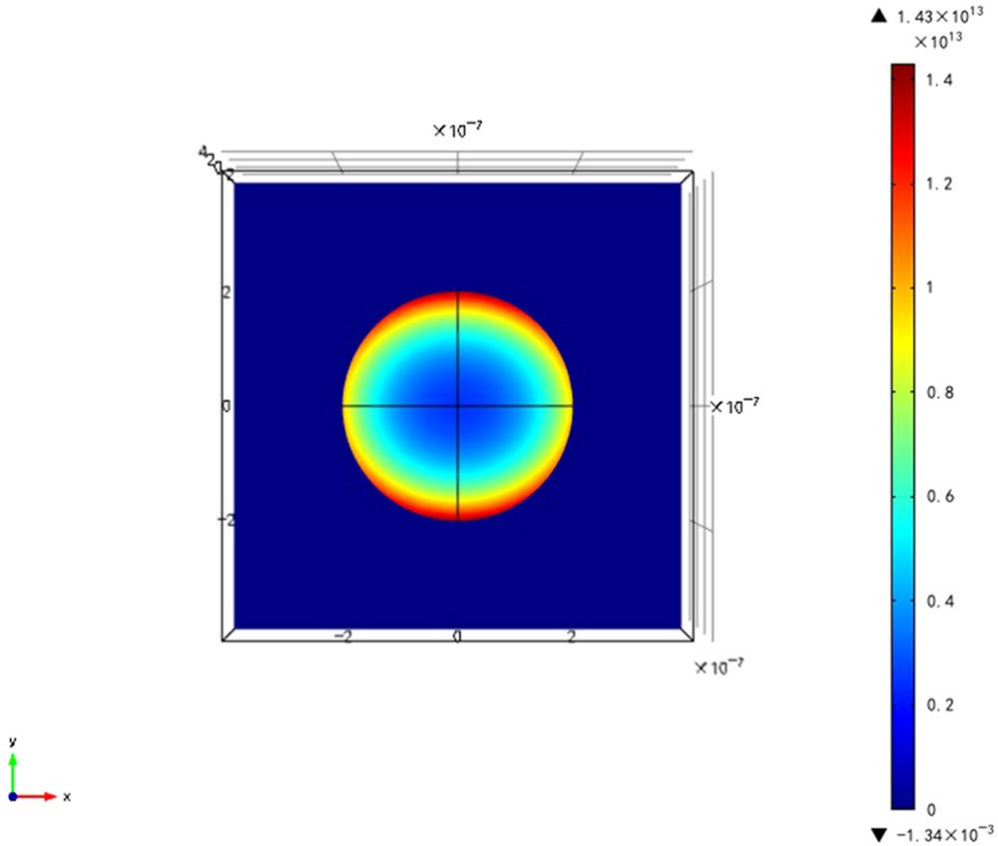
The electric field intensity of Au@Cu_2_S nanocrystals at 1300 nm in the absorption peak.

Overall, at the wavelength of 1300 nm, we simulated the absorption intensity with Au@Cu_2_S nanocrystals, as shown in Fig. 4. We can see after inducing the excitation of nanoparticles at 1300 nm, the electric field intensity of the surface of Au@Cu_2_S was powerful, which suggests the nanoparticle exhibited LSPR absorbance at 1300 nm. Therefore, the result indicates that Au@Cu_2_S nanocrystals have an obvious LSPR absorbance in the absorption peak we acquired by using UV-VIS-NIR scanning spectrophotometer.

**Figure 5:**
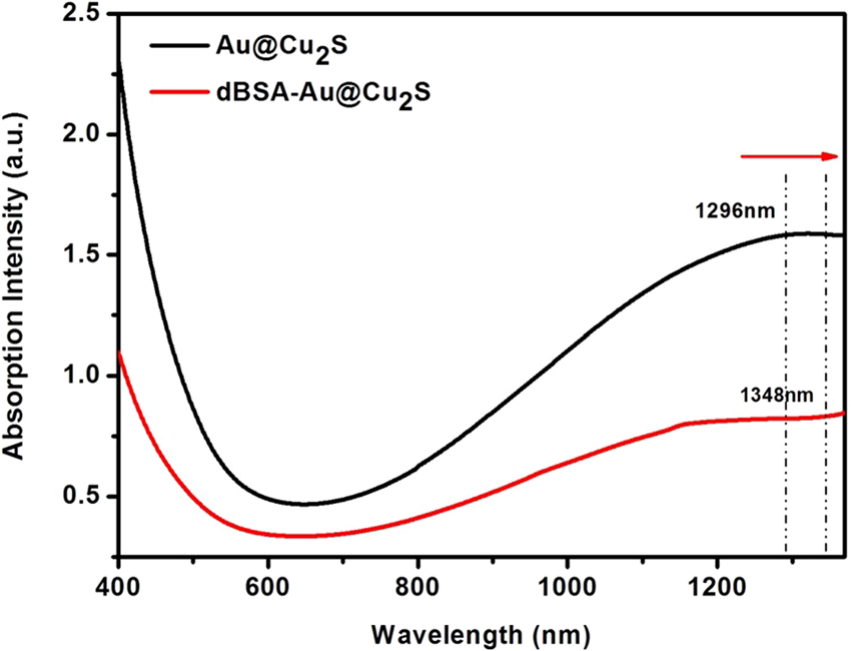
UV–vis absorption spectrum and the peak of Au@Cu_2_S (1296 nm) and dBSA-Au@Cu_2_S (1348 nm).

Figure 5 shows that when the absorption spectrum of Au@Cu_2_S and dBSA- Au@Cu_2_S were at the same concentration, the dBSA acted as a stabilizer to improve the stability and biocompatibility further. We could see that there was a redshift in the dBSA-Au@Cu_2_S absorption, and it was due to the change in the dielectric constant of the medium surrounding the nanocrystals^37,38^.

**Figure 6:**
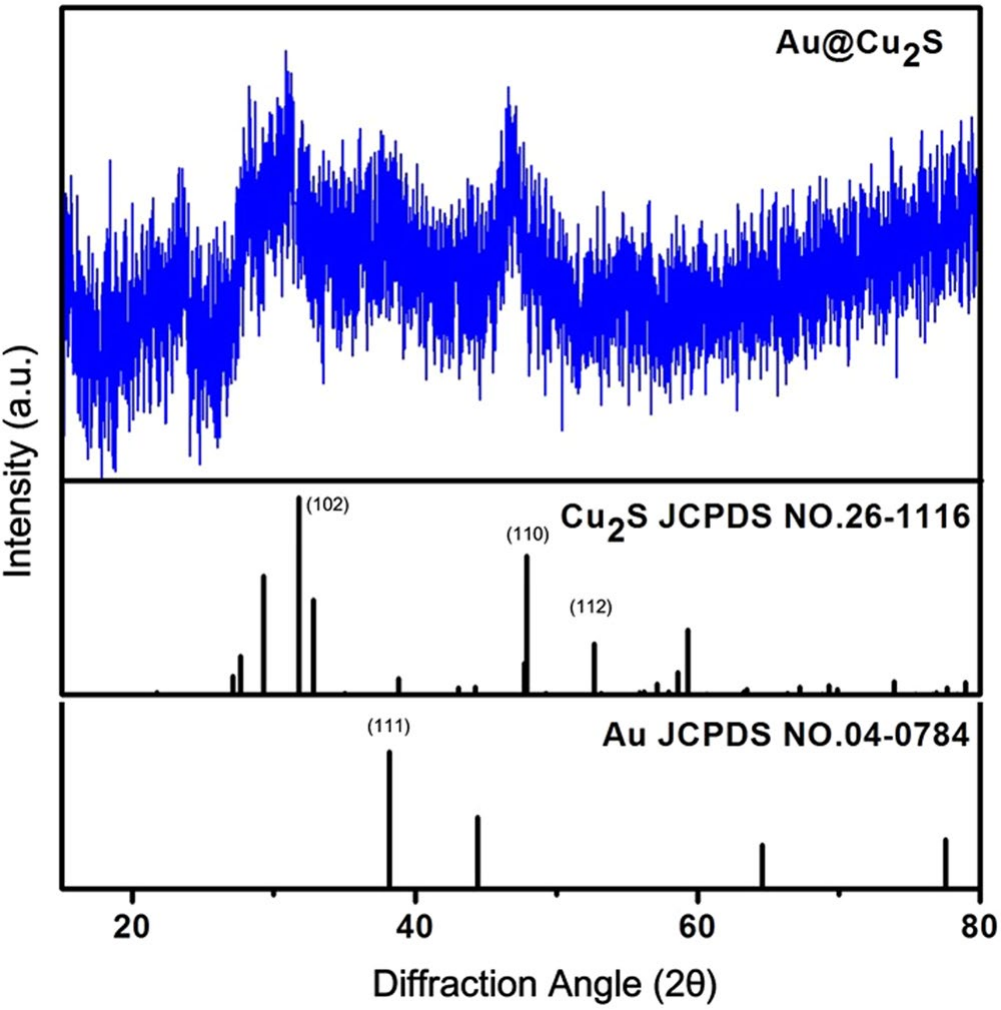
X-ray diffraction (XRD) spectrum of Au@Cu_2_S nanocrystals.

The XRD pattern of the nanocrystals obtained by Au@Cu_2_S as shown in Fig. 6 exhibited a significantly reduced at wurtzite peak, which indicated that the incorporation of Cu and Au might suppress the growth of the wurtzite plane in the Au@Cu_2_S nanocrystals. This further demonstrated the importance of the composition in determining the crystal structure. And to further verify the Cu_2_S phase, we have test the X-ray photoelectron spectroscopy (XPS) of Cu_2_S, as shown in Figure S3(a) represents the XPS high resolution scan of Cu 2p in Cu_2_S with peaks position at 951.3 eV (Cu 2p_1/2_) and 931.5 eV (Cu 2p_3/2_), the binding energies of the samples reveal that copper is present as Cu_2_S from which is in agreement with earlier report^39^.

**Figure 7:**
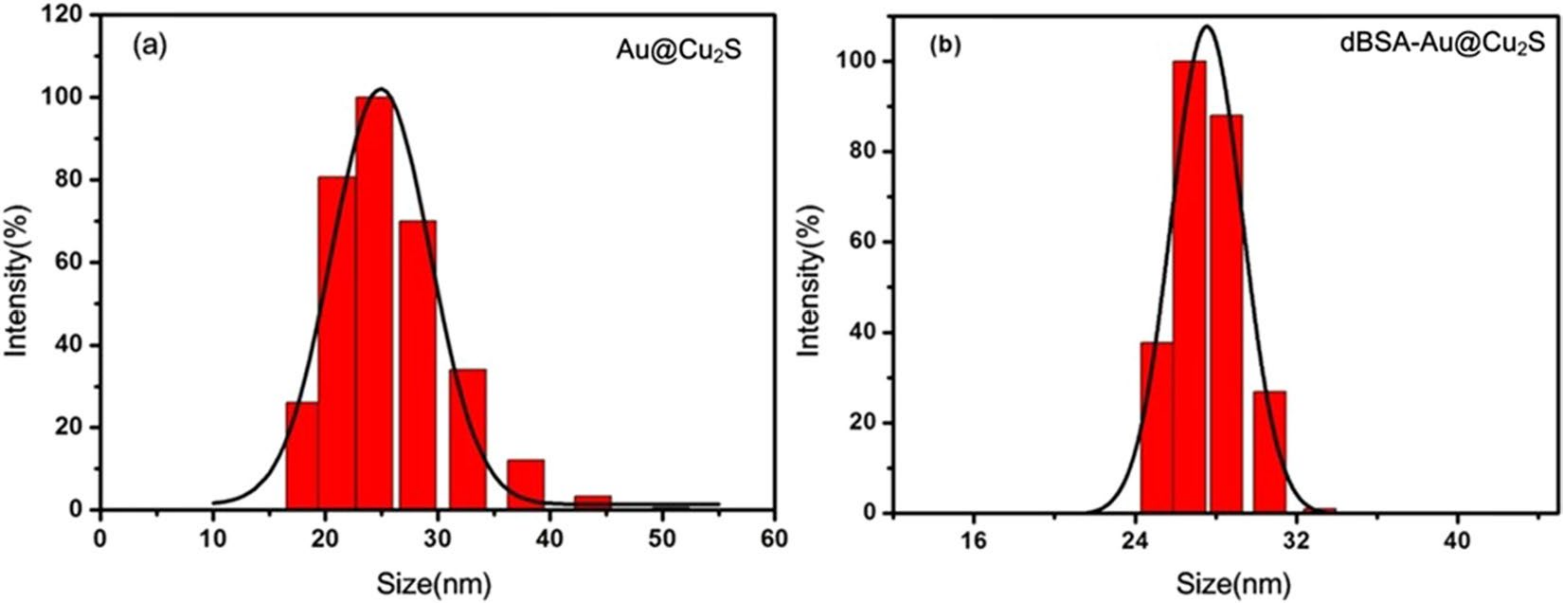
The hydrodynamic diameter distribution of (**a**) Au@Cu_2_S and (**b**) dBSA-Au@Cu_2_S.

It was important to make sure that there was no severe aggregation after the process of dBSA bioconjugation. The hydrodynamic sizes of two samples were measured by using the dynamic light scattering (DLS) method, as shown in Fig. 7(a,b), which showed that the particles were respectively ~26 nm, and ~24 nm with a small size distribution, but larger than that found from the TEM data because the surface of the particles was covered by water molecules. Also, we monitored the distribution of Au@Cu_2_S and dBSA-Au@Cu_2_S in DI water (with pH values 4, 7 and 9) and PBS for 100 hours, the results are shown in the supplementary information Figure S1. We can see the dBSA-Au@Cu_2_S is much more stable in solutions with pH 4, 7 and 9 when compared to Au@Cu_2_S. Also, we tested the stability of the dBSA-Au@Cu_2_S dissolved in PBS (pH = 7.2), the result shows the dBSA-Au@Cu_2_S is very stable in the PBS solution. This result indicated that dBSA-Au@Cu_2_S particles were well dispersed.

**Figure 8:**
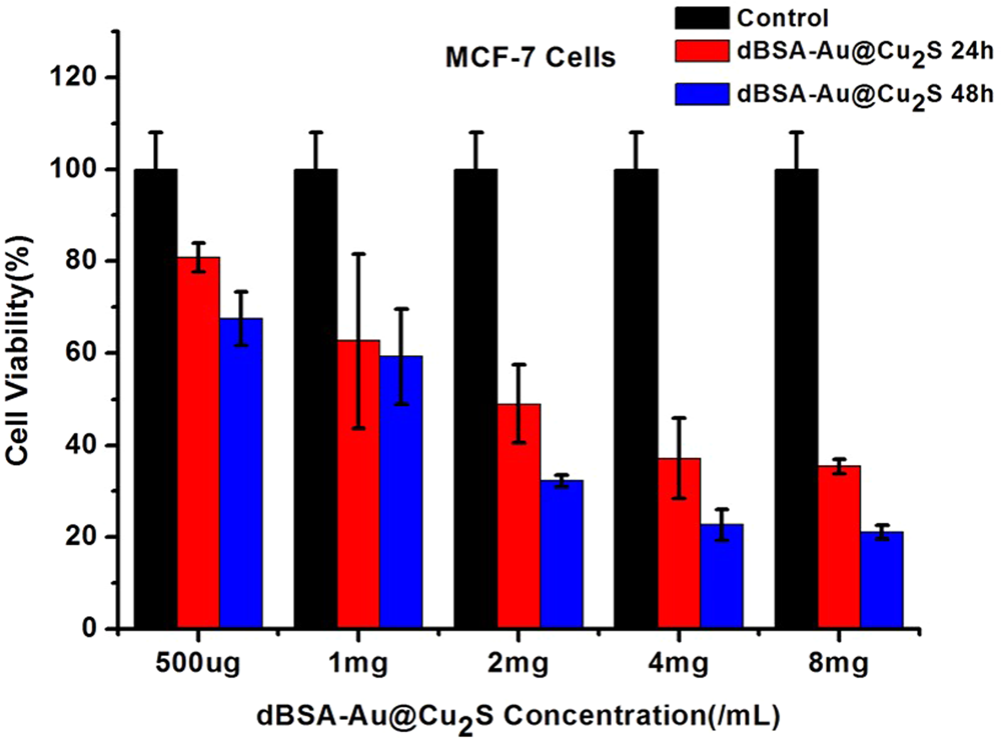
Relative cell viability of MCF-7 breast carcinoma treated with varying concentrations of dBSA-Au@Cu_2_S in 24 h and 48 h.

Figure 8 shows the cell viability of MCF-7 breast cancer cells after treating them with dBSA-Au@Cu_2_S for respectively 24 and 48 hours under different concentrations from 45 μgml^−1^ to 720 μgml^−1^ (20 μl of nanoparticles and 200 μl of cell culture medium). Before we test the cytotoxicity of dBSA-Au@Cu_2_S, we have done the MCF-7 breast cancer cell imaging study, as shown in Figure S2, we have demonstrated this nanocrystal can go into the cells by cytophagy. Then in cytotoxicity test, the cells treated with dBSA-Au@Cu_2_S nanocrystals have maintained more than 80% cell viability even at concentrations as high as 45 μgml^−1^ for 24 hours. After 48 hours, the cells treated with dBSA-Au@Cu_2_S nanocrystals kept higher than 70% cell viability at the same concentration level. And to further verify the biocompatibility of our nanocrystal, we also did the cell viability study of RAW264.7 macrophage cells, the result as shown in Figure S4, which indicated that dBSA-Au@Cu_2_S nanocrystals were low in toxicity and were biocompatible.

In recent years, due to the absorption wavelength belonging to the “therapy window”^40–42^, the more metal nanocrystals have been used as a contrast-enhancer for clinical detection depending on their strong absorption in NIR field^43–45^. To confirm the stability and biocompatibility, further *in vivo* imaging measurements were performed with dBSA-Au@Cu_2_S based on OCT imaging detection. Optical coherence tomography (OCT) uses low coherence interferometry to obtain micron-resolution images of scattering samples. As we know, the OCT detection signal effect applies the backscattered light intensity of contrast agents, and the OCT light source is 1300 nm, which almost matches with the absorption of dBSA-Au@Cu_2_S. A match of the absorption wavelength will lead to the LSPR, which can enhance the scattering of the sample. Additionally, Au can increase the scattering signal. Therefore, the OCT signal can be amplified^46^.

**Figure 9:**
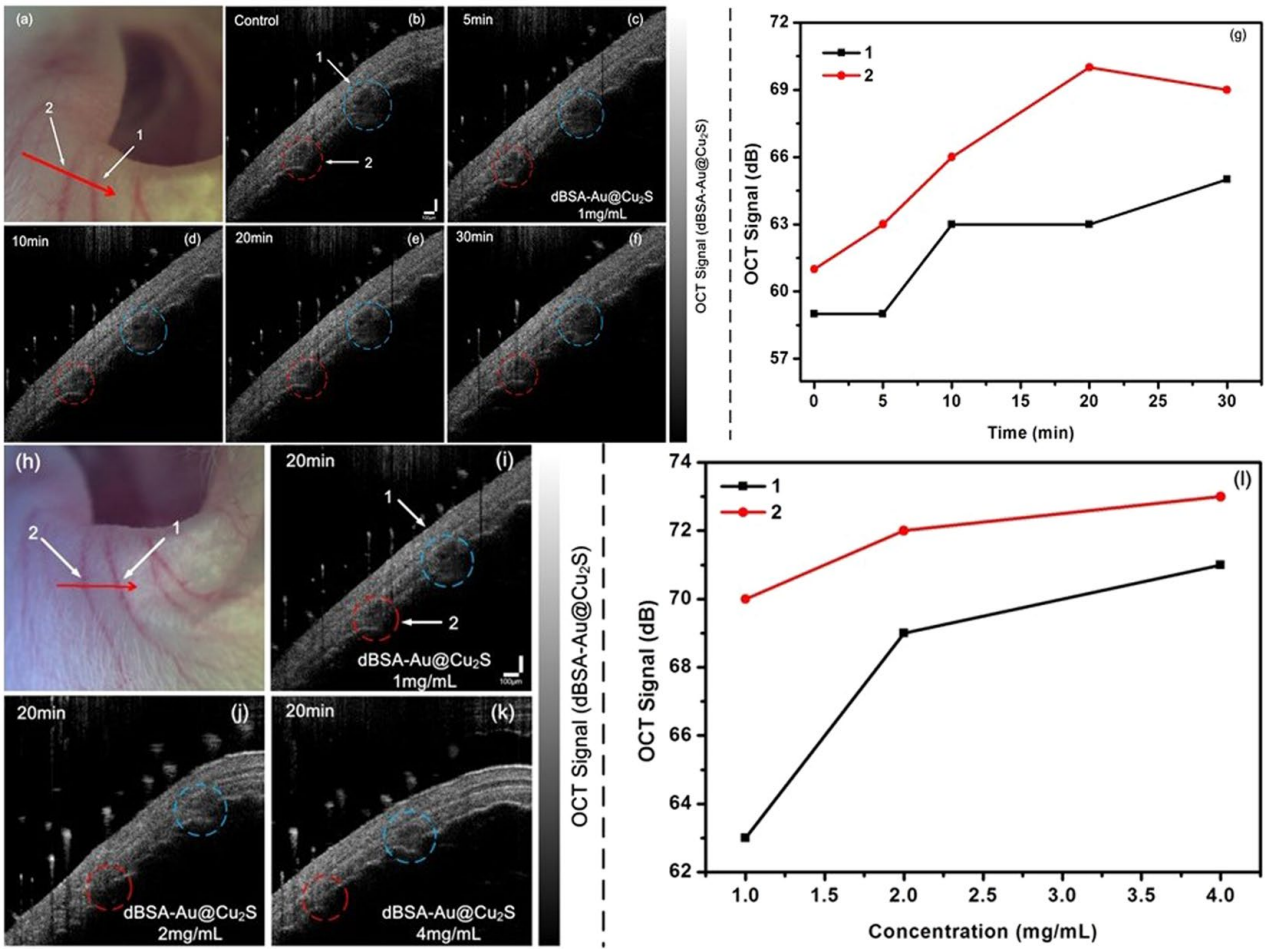
Photograph of mouse left ear pinna, (**a**) and (**h**) are images of the blood vessel under OCT detection, the red line denotes the location of the acquired B-scan. (**b**–**f**) The OCT intensity images of before and after 5, 10, 20, 30 mins as intravenous nanocrystals by circulation, respectively. (**g**) Value of OCT signal changing with the nanocrystal cycling in the blood vessels of mice. (**i**–**k**) OCT intensity images of different intravenous nanocrystal concentrations (1 mg/mL, 2 mg/mL and 5 mg/mL, respectively) by the same circulation time. (**l**) The OCT intensity of the intravenous nanocrystal concentration enhances in blood vessels.

Figure 9 shows the *in vivo* experiments of a living mouse with a tail injection. As shown in Fig. 9 (a), in which 1 and 2 were the blood vessels of the left pinna with 1 mg/mL dBSA-Au@Cu_2_S and were circulated for 5 and 30 minutes, respectively. The OCT acquired the signal of the nanocrystals by circulation to the left ear pinna after 5 minutes, the time of accumulation for the maximal nanocrystal concentration in the ear was observed at 20 minutes, and then the concentration decreased as the nanocrystals diffusion was in circulation for 30 minutes. The OCT signal shows the data were linear with the circulation time due to Au@Cu_2_S nanocrystals encapsulated in dBSA, which enhanced the circulation time in blood. With the increase in the cycle time, it was hypothesized that the sample would gather in the ear, and the LSPR signal from the sample will lead to an increase in the signal.

To further confirm, we obtained OCT images for different concentrations (from 1 mg/ml to 4 mg/ml) of the nanocrystals at the same time, as shown in Fig. 9(h–k). The signals of the two blood vessels marked by the red and blue circulars continued to accumulate in 20 minutes for the circulation in the left ear pinna. As with the *in vivo* experiments, we determined that the nanocrystals could lead to a change in the OCT signal. The signal increased as the concentration of the nanocrystals increased. This result matched with the previous reports^47^. These results demonstrate that Au@Cu_2_S can be applied as a near-infrared contrast medium and may have many uses in the *in vivo* bioimaging study.

## Conclusion

We have found that Au@Cu_2_S core/shell-structured nanocrystals were an excellent contrast-enhancer for OCT imaging. Using dBSA as the encapsulation micelles, conjugated with Au@Cu_2_S nanocrystals. The colloidal stability and the low cytotoxicity, as well as the biocompatibility of the encapsulated nanocrystals, were observed. Additionally, through the OCT test, these nanoprobes can serve as diagnostic tools for *in vivo* bioimaging. This study has revealed that the metal core/shell structured-nanocrystals are a strong candidate for a contrast-enhanced for bioimaging study. We are currently extending our research to focus on the drug loading efficiencies and clinical cancer therapies by using NIR absorption of the metal nanocrystals.

## Methods

### Chemicals

Gold(III) chloride trihydrate (HAuCl_4_•3H_2_O, 99.99%), Sodium borohydride (NaBH_4_, 98%), Copper(I) chloride (CuCl, 99.999%), Sodium hydroxide (NaOH, 50%) were purchased from Alfa Aesar. Sodium sulfide (Na_2_S, 62~65%) was purchased from Acros Organics. Glutathione (GSH, 97%), albumin from bovine serum (BSA, ≥98%) were purchased from Sigma-Aldrich. All chemicals were used as received without further purification. Deionized (DI) water used in all the studies was purified by a Milli-Q water purification system.

### Synthesis of Au@Cu_2_S

The synthesis method we applied is the aqueous-phase method. Compared with the quantum dots proposed in our previous article, the nanoparticles we used in previous were synthesized in oil phase^48^. And during OCT biometric imaging experiment, we need to transfer the phase of nanoparticle from the oil phase to aqueous phase. But the purpose of our group in the development of nanoparticles is to better use in biological experiments and continuously improve its synthesis method, to facilitate biological imaging detection methods. So, in this paper, aqueous-phase Au-Cu_2_S were prepared using a new one-step synthesis method. In the one-step synthesis, 100 mL of ultrapure water was heated to 90 °C in a three-neck flask (150 mL) fitted with a valve and deaerated by bubbling with 99.99% nitrogen under magnetic stirring for 30 min. Glutathione (2 mmol, 614.56 mg) was injected, and the solvents were stirred by continuously purging with nitrogen for 10 min. Copper(I) chloride (1 mmol, 99 mg) powder and 400 µL of HAuCl_4_ (10 mM) were rapidly added to the flask while keeping positive nitrogen pressure to prevent air from entering. After refluxing for 1 hour at 90 °C, the pH of the formed emulsion was adjusted to 10.0 by adding a 0.5 M solution of sodium hydroxide. The solution became light brown and transparent after 1 min. Then, NaBH_4_ (78.4 mg) was added to the solution for the reduction HAuCl_4_ to Au nanoparticles. The solution came to equilibrium. The solution of sodium sulphide was obtained by the dissolution of 59.55 mg (0.25 mmol) of sodium sulphide in 3 mL of ultrapure water. After another 5 min, this sodium sulphide solution was injected into the reaction mixtures all at once. The colour of the solution turned light brown after 5 min, indicating the formation of the Au-Cu_2_S. The colour continued to deepen and gradually turned dark brown after 10 min.

### Synthesis of dBSA-Au@Cu_2_S

Synthesis of dBSA, 200 mg of BSA and 6 mg NaBH_4_ were added to 5 mL ultrapure water, which was then magnetically stirred at 70 °C for 1 h. The hydrogen was removed, and the concentration of dBSA was 40 mg/mL. The synthesis of dBSA-Au@Cu_2_S, 2 mL of Au@Cu_2_S (2 mg/mL) and 440 µL dBSA(40 mg/mL) were mixed and stirred at 70 °C for 30 min, the dBSA-Au@Cu_2_S was synthesized by this method.

### Characterization of nanocrystals structure

DLS technique: the diameter was detected by the DLS technique using a Malvern Nano ZS90 Zeta sizer potential. Transmission electron microscopy (TEM): the size and morphology of the NCs were determined by TEM using an FEI microscope at a working voltage of 200 kV. Selected area electron diffraction (SAED) patterns and high-resolution TEM were obtained in the same instrument. X-ray diffraction: powder XRD (Bruker Ultima IV with Cu Kα X-ray) was employed to characterize the crystal structure of the samples. The samples were prepared by drop-casting a highly concentrated (10 mg/ml) Au@Cu_2_S solution onto glass. UV–vis spectroscopy: the optical absorbance of the samples was measured using an Agilent Cary-5000 UV-vis-NIR scanning spectrophotometer.

### *In vitro* bioimaging study

MCF-7 Breast cancer cells (American Type Culture Collection) were cultured with Dulbecco’s Modified Eagle’s Medium (DMEM, Hyclone), supplemented with 10% fetal bovine serum (FBS, Hyclone), penicillin (100 µg ml^−1^, Gibco) and streptomycin (100 µg ml^−1^, Gibco) in a humidified environment (37 °C, 5% CO_2_). Before treating with QDs, cells were seeded onto cover glasses in a 6-well plate with DMEM medium. The prepared FITC-dBSA-Au@Cu_2_S formulations were then diluted with PBS buffer (pH = 7.2) solution to a concentration of 500 μg/mL. Next, the cells were treated with the QD formulations for 4 h. After 4 h of incubation, the treated cells were washed with PBS buffer for three times. Leica DMI3000 inverted Microscope with a 10 × lens was used for cell imaging study, and the excitation wavelength is 477 nm, the emission wavelength is 530 nm.

### Evaluation of *in vitro* cytotoxicity

For each MTS assay, 24 culture wells of MCF-7 breast cancer cells were prepared. Seven sets were treated with different concentration of Au@Cu_2_S, and one set was used as the control. The complete assay was performed in triplicate, and the results were averaged. Various concentrations of the Au@Cu_2_S formulation ranging from 500 μg/mL to 8 mg/ml were added to each well and subsequently incubated with MCF-7 breast cancer cells for 24 and 48 hours at 37 °C under 5% CO_2_.

### Small animal study

Four-week-old Kunming mice were purchased from the experimental animal center of Jilin University. The animal experiments were carried out under the animal protocol approved by the School of Life Science, Jilin University. The animal housing area was maintained at 24 °C with a 12 hour light/dark cycle, and the animals were fed ad libitum with water and standard laboratory food. All operations were carried out by the National Standard of Animal Care and Use Procedures (20080820). The mice were administered the nanoparticle formulations in 1^x^PBS through subcutaneous injection.

### OCT Test

The OCT test was carried out on a TELESTO (Thorlabs, USA) with a 1300 nm light source, and the in-depth and lateral ranges are 2.5 mm and 5.5 μm, respectively. The line rate is 76 kHz with a sensitivity of 111 dB.

## Electronic supplementary material

Supplementary information
