# Supplementary material for: Optimizing the Synthesis of Core/shell Structure Au@Cu2S Nanocrystals as Contrast-enhanced for Bioimaging Detection
Source: Sci Rep. 2018 Jun 11;8:8866. doi: 10.1038/s41598-018-27015-x (PMC5995884; doi:10.1038/s41598-018-27015-x)
Supplement: Supplementary file 1 — Supplementary information [file 41598_2018_27015_MOESM1_ESM.pdf]

Electronic Supplementary Information

Optimizing the synthesis of core/shell structure Au@Cu_2S nanocrystals as contrast-enhanced for bioimaging detection

Liwei Liu[1], Siyi Hu [2], Yue Wang[3], Shaozhuang Yang[1], Junle Qu[1*]

[1] Key Laboratory of Optoelectronic Devices and Systems of Guangdong Province, College of Optoelectronic Engineering, Shenzhen University, Shenzhen, Guangdong Province, 518060, P.R. China

[2] CAS Key Laboratory of Bio-Medical Diagnostics, Suzhou Institute of Biomedical Engineering and Technology, Chinese Academy of Sciences, Suzhou, Jiangsu, 215163, P.R.China

[3] International Joint Research Center for Nanophotonics and Biophotonics, School of Science, Changchun University of Science and Technology, Changchun, Jilin Province, 130022, P.R. China

Email: jlqu@szu.edu.cn

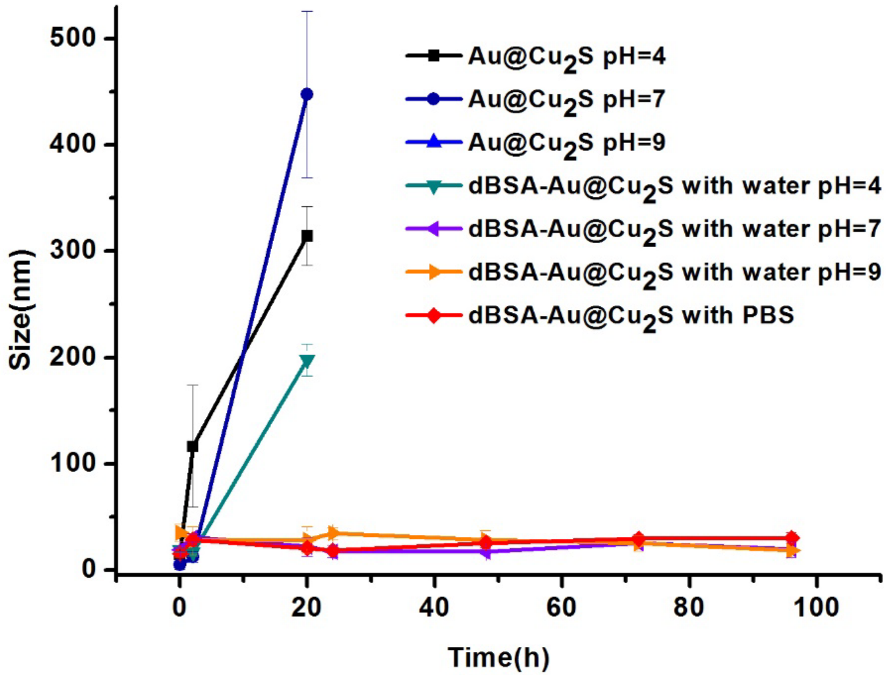

Figure S1. Comparison of colloidal stability between Au@Cu_2S (dissolved in DI water) and dBSA-Au@Cu_2S (dissolved in DI water and PBS) at different pH values (4, 7, 9) for 100 hours.

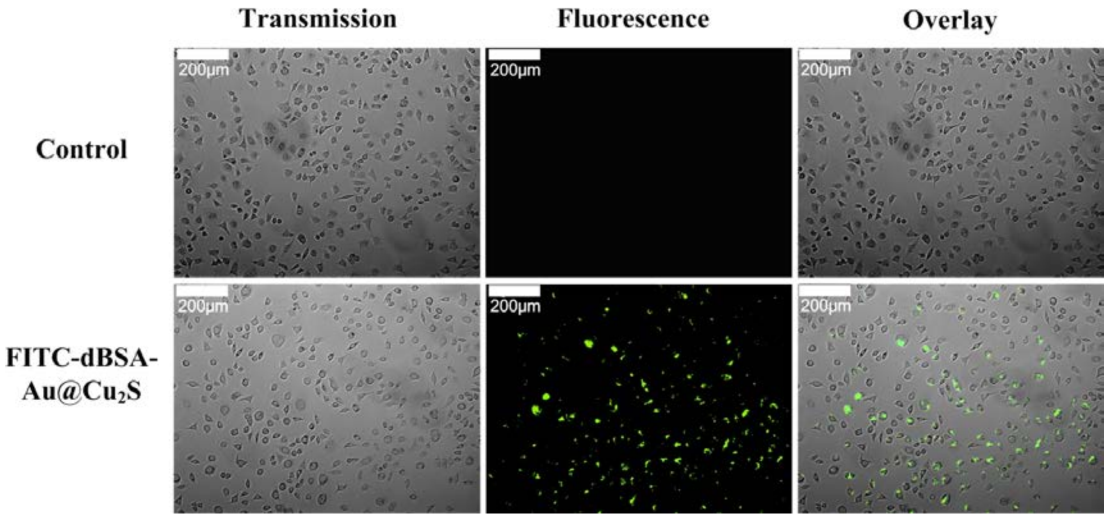

Figure S2. Microscopic images of MCF-7 breast cancer cells labeled with FITC-dBSA-Au@Cu_2S.

MCF-7 Breast cancer cells (CAmerican Type Culture Collection) were cultured with Dulbecco's Modified Eagle's Medium (DMEM, Hyclone), supplemented with 10% fetal bovine serum (FBS, Hyclone), penicillin (100μg ml^{-1}, Gibco) and streptomycin (100μg ml^{-1}, Gibco) in a humidified environment (37°C, 5% CO_2). Before treating with QDs, cells were seeded onto cover glasses in a 6-well plate with DMEM medium. The prepared FITC-dBSA-Au@Cu_2S formulations were then diluted with PBS buffer (pH=7.2) solution to a concentration of 500μg/mL. Next, the cells were treated with the QD formulations for 4h. After 4h of incubation, the treated cells were washed with PBS buffer for three times. Leica DMI3000 inverted Microscope with a 10x lens was used for cell imaging study.

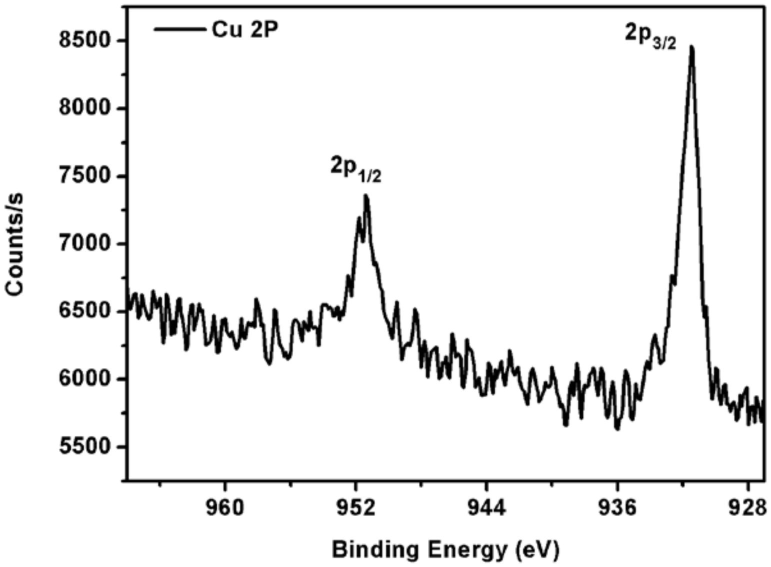

Figure S3. X-ray photoelectron spectroscopy (XPS) of Cu2S, the high resolution scan of copper 2p.

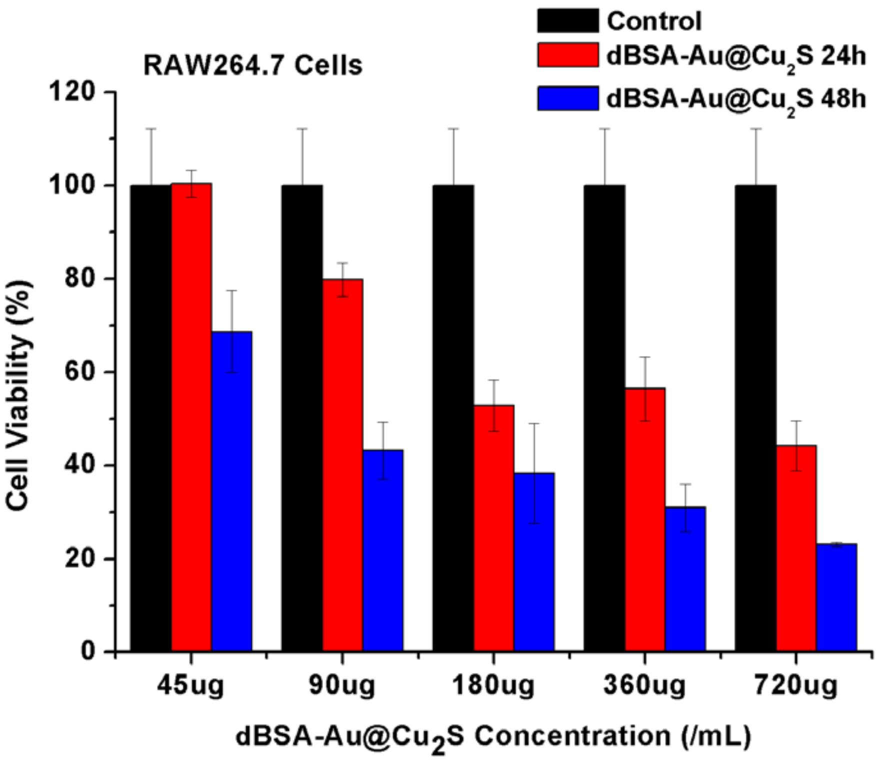

Figure S4 Relative cell viability of RAW264.7 Cells treated with varying concentrations of dBSA-Au@Cu_2S in 24 h and 48 h.

The cell viability of RAW 264.7 cells after treating them with dBSA-Au@Cu_2S for respectively 24 and 48 hours under different concentrations from 45μgml^{-1} to 720μgml^{-1} (20μl of nanoparticles and 200μl of cell culture medium).
